# From passion to pressure: exploring the realities of the teaching profession

**DOI:** 10.3389/fpubh.2025.1505330

**Published:** 2025-03-07

**Authors:** Joy C. Nwoko, Emma Anderson, Oyelola A. Adegboye, Aduli E. O. Malau-Aduli, Bunmi S. Malau-Aduli

**Affiliations:** ^1^College of Medicine and Dentistry, James Cook University, Townsville, QLD, Australia; ^2^College of Public Health, Medical and Veterinary Sciences, James Cook University, Townsville, QLD, Australia; ^3^Menzies School of Health Research, Charles Darwin University, Casuarina, NT, Australia; ^4^School of Environment and Life Sciences, The University of Newcastle, Callaghan, NSW, Australia; ^5^School of Medicine and Public Health, The University of Newcastle, Callaghan, NSW, Australia

**Keywords:** teaching profession, teacher occupational well-being, psychosomatic symptoms, stress and burnout, self-efficacy, job satisfaction, social support

## Abstract

**Introduction:**

Teacher retention, workload, and the intention to leave the profession have become growing concerns in education, highlighting the need for a holistic approach to teacher occupational well-being.

**Methods:**

This study employed a sequential explanatory mixed methods design to investigate factors influencing teacher well-being. A cross-sectional quantitative survey (*n* = 247) examined teachers’ perceptions of occupational well-being, while phenomenological qualitative interviews (*n* = 21) explored their workplace experiences. Using the OECD teacher occupational well-being framework, quantitative and qualitative data were integrated to identify key determinants of well-being and potential strategies for improvement.

**Results:**

Findings revealed that teachers with strong self-efficacy and social support experienced higher job satisfaction and fewer psychosomatic symptoms, whereas increased stress levels led to greater health-related issues. Male teachers reported fewer psychosomatic symptoms than female teachers, while experienced teachers exhibited higher stress levels. Early-career teachers and junior-grade classroom teachers were more likely to consider leaving the profession, with larger class sizes contributing to greater stress and burnout. Workplace stress, student intimidation, and verbal abuse were positively associated with psychosomatic symptoms, while addressing parent or guardian concerns correlated with improved cognitive and social well-being. Lack of support was a major contributor to stress, burnout, and job dissatisfaction, whereas strong social support networks alleviated these issues.

**Conclusion:**

The study underscores the importance of ongoing leadership support and well-being-centered policies in fostering teacher occupational well-being and improving retention, particularly among early-career educators. These findings provide valuable insights for school administrators, policymakers, and educators to develop targeted strategies that create a supportive and sustainable teaching environment.

## 1 Introduction

Increasing interest in teacher well-being has emerged globally due to concerns about the teaching profession and student outcomes, especially following the COVID-19 pandemic (1–4). The term “Well-being” is a broad and complex concept that may relate to people’s happiness, quality of life, as well as physical or mental health (5). Across different occupations, there is a positive correlation between work engagement and occupational well-being (6). Thus, teacher work engagement is a crucial component of their occupational well-being (1).

There is also a growing recognition of the importance of providing all students with access to highly engaged and motivated teachers (7, 8). Evidence suggests that teacher and student engagements are reciprocal, with high teacher engagement positively impacting student engagement and achievement (3). This reciprocal relationship underscores the importance of addressing teacher engagement to be able to understand and mitigate attrition from the profession, a trend often linked to low engagement and job dissatisfaction (9, 10).

The decision to leave teaching has been recognized as a significant problem in education systems worldwide (11). For instance, the teacher turnover rate in Europe was reported at 28% across 19 countries (12), while 40% of Chinese teachers expressed willingness to leave the profession for other opportunities (13). In Australia, a quarter of teachers intended to leave the profession before retirement, citing stress and its impact on well-being as reasons for leaving (14). Similar trends were observed in Finland, where 50% of teachers intended to leave within 5 years, citing workload and system complexity as reasons (11). These findings highlight the global nature of the issue and the need for targeted interventions. This is particularly important within the Australian context where teachers work longer hours compared to their international counterparts and are therefore exposed to significant occupational stress (8).

Teacher occupational stress, a response to perceived challenging demands, negatively impacts psychological and physical well-being (15, 16). Stressors often originate from the work environment and conditions (17). Teaching is inherently demanding and challenging (18, 19), and unmanaged occupational stress can lead to mental health problems, burnout, and decision to leave the profession (20–22). Factors such as workload, lack of administrative support, and perceived lower social status of the profession diminish, teacher occupational well-being and retention (23–26). Studies globally, including in Australia, indicate that teachers are at high risk of stress and burnout due to their jobs (18, 27–30).

Long before the pandemic, research studies have reported increasing teacher stress and declining well-being (31). For example, 51% of American teachers reported feeling great stress several days a week in 2012, up from 36% in 1985 (32). This trend continued during and after the pandemic, with teachers in Western countries, including Australia, England, Canada, and the USA, reporting overwhelming responsibilities (33). In Australia, data collection and reporting were major sources of stress in 2021 (34). However, findings on teacher stress are inconsistent. Some studies found primary school teachers experiencing greater stress and burnout than high school teachers (21, 28, 29, 35, 36), while others found no differences (37) or argued that burnout risk does not vary with experience or teaching level (38, 39). Nevertheless, Carroll et al. (29) reported that primary school teachers were significantly more stressed due to additional administrative and assessment processes. The political discourse often portrays effective teachers as selfless and resilient, which may further contribute to stress (40, 41).

To enhance support intervention strategies for primary school teachers, it is crucial to examine factors that influence their occupational well-being, teaching engagement, and motivation to stay in the profession. Workload is a frequently cited source of stress (29). Attracting and retaining quality teachers and ensuring greater instructional productivity, work engagement, and occupational commitment requires a deeper understanding of the factors that impact their work and productivity (42).

The intensification of teaching in primary schools particularly affects primary school teachers, who have more face-to-face time with students and more parent contact compared to other teacher types (43, 44). Teachers often experience heightened levels of workload and stress due to the continuous supervision and attention required by young children. Managing younger children necessitates substantial energy and patience, contributing to exhaustion and a high degree of emotional labor (28). In the Australian context, teachers work longer hours compared to their international counterparts, averaging 43 h per week (29, 45), which is about 5 h more than in many other countries (8, 45). Additionally, administrative tasks rather than classroom teaching hours are a significant source of stress for Australian teachers (29). Therefore, with a focus on the Australian context, this study aims to investigate the association between teachers’ occupational well-being and their stress and burnout levels, teaching engagement, and motivation to stay in the profession.

### 1.1 Theoretical framework for this study

This study adapted the Organization for Economic Co-operation and Development (OECD) teacher occupational well-being framework as a conceptual framework and the recently developed teacher occupational well-being questionnaire for the 2021 Programme for International Student Assessment (PISA) to guide this research. The framework guides the understanding of the core components of teachers’ occupational well-being as well as the working conditions that shape teachers’ occupational well-being.

The OECD conceptual framework depicts that teacher occupational well-being encompasses four major dimensions of the teaching role, including cognitive, subjective, physical & mental, and social aspects (46). Teachers’ cognitive well-being refers to the self-efficacy skills and abilities that the teacher needs to work effectively (47). Teachers’ self-efficacy impacts their enthusiasm, commitment to teaching, job satisfaction, and their professional practice (48). The Subjective dimension of teachers’ well-being relates to the evaluations that teachers make concerning their experience in their teaching job and how satisfied or fulfilled they are with their job (45). Job satisfaction is a sense of fulfillment that teachers get from working (49), and is positively related to teacher occupational well-being and commitment to teaching, self-efficacy, and motivation (45, 50). The physical and mental dimension of teachers’ well-being relates to teachers’ health (46). The stress that teachers experience in their job role may result in complaints and psychosomatic symptoms (51). By their profession, teachers are required to relate with all stakeholders including students, colleagues, parents, principals, support staff, specialists, and consultants (52, 53). Therefore, social well-being in this context relates to the depth and quality of social interactions between teachers and stakeholders (46). Support from management or lack of it, issues with parents, and students’ misbehavior are some of the issues that can affect teachers’ social well-being (54). The framework also considers the inward outcomes of these four dimensions in terms of their impact on teachers’ stress levels and motivations to continue with the profession as well as the outward outcomes which refer to the classroom processes and school factors.

This study is part of a larger project that aims to explore the associations between mainstream Australian primary school teachers’ well-being and working conditions at both system and school levels as well as the attributable inward and outward outcomes. This part of the research focuses on the inward outcomes of the four teacher occupational well-being dimensions in terms of how they are impacted by teachers’ stress and burnout levels and their motivations to continue with the profession.

The two questions addressed in this study are:

1. What is the influence of stress levels and motivation to leave the profession on teachers’ occupational well-being?
2. How do teachers perceive the impact of their professional experiences on their decision to remain in or leave the teaching profession?

## 2 Methodology

### 2.1 Study design

The present study used a pragmatic-inquiry-based sequential explanatory mixed methods design (55), which utilized both quantitative and qualitative research methods to explore the relationship between the teacher’s occupational well-being, teaching engagement, stress levels, and motivation to stay in the profession. Mixed methods research allows for complete and synergistic utilization of data to validate findings from qualitative and quantitative data sources (56). Triangulation of findings from the two research methods aided the uncovering of the best possible explanations for the observed phenomenon (57, 58).

### 2.2 Quantitative phase

The quantitative phase of the study was conducted first using a questionnaire to capture teachers’ perception of their occupational well-being, teaching engagement, stress levels, and motivation to stay or leave the profession. Findings from the quantitative phase guided the development of the interview questions for the qualitative phase. Permission was obtained from the authors (46) before using the OECD questionnaire.

#### 2.2.1 Participant recruitment and sampling

Participants were recruited through convenience sampling, focusing on primary school teachers in Australia. A national approach was used in recruiting participants.

Recruitment efforts included educational forums, educator-focused social media platforms, and snowball sampling (59). All potential participants received comprehensive information about the study’s objectives, the voluntary nature of their involvement, and the ethical protocols, including confidentiality measures implemented. Data was collected through Qualtrics which is an anonymous online survey platform.

#### 2.2.2 Design and data collection

The quantitative phase of this study answered the first research question, and it involved the utilization of a cross-sectional survey of mainstream primary school teachers to examine the demographic variables (e.g., age, gender, years of experience) that influence teachers’ occupational well-being, stress levels and motivation to stay in or leave the profession. A cross-sectional design enables the examination of multiple variables and their relationships simultaneously (60). This is essential for a comprehensive analysis of the various factors influencing teachers’ occupational well-being and professional decisions.

#### 2.2.3 The survey instrument

This study adapted the OECD teacher occupational well-being framework questionnaire developed by Viac and Fraser (46) for data collection. The adapted questionnaire had 17 questions that focus on inward outcomes—stress and burnout, and motivation to leave the profession. The indicators were tested against the four core dimensions of teacher occupational well-being. Thirteen additional questions were used to capture participants’ demographic variables (see Appendix 1).

The four core dimensions of teacher occupational well-being included cognitive, subjective, social as well as physical and mental dimensions.

##### 2.2.3.1 Cognitive dimension

Twelve questions measured teacher self-efficacy in relation to three distinct competencies that teachers require while teaching in the classroom: efficacy in classroom management (*n* = 4), efficacy in instruction (*n* = 4), and efficacy in student engagement (*n* = 4). Teachers rated all items on a four-point scale ranging from 1 (not at all) to 4 (a lot).

##### 2.2.3.2 Subjective dimension

Job satisfaction was assessed using nine questions in relation to how fulfilled or satisfied teachers were with their jobs. The questions measured two aspects of teachers’ job satisfaction: Job satisfaction with the profession (*n* = 5), and the current working environment (n = 4). Teachers rated all items on a four-point scale ranging from 1 (strongly disagree) to 4 (strongly agree).

##### 2.2.3.3 Physical and mental dimension

Psychosomatic symptoms were assessed using ten questions that measured ten (*n* = 10) health related complaints that can affect teachers’ occupational well-being. Teachers rated all items on a five-point scale ranging from 1 (Never or almost never) to 5 (Every day or almost every day).

##### 2.2.3.4 Social dimension

Fifteen questions measured social relationship/support in two distinct social interactions that transpire between the teacher and other stakeholders in the school environment: social support from colleagues (*n* = 5), social support from principal (*n* = 5) and teacher-student relations and support to students (*n* = 5). Teachers rated all items on a four-point scale ranging from 1 (strongly disagree) to 4 (strongly agree).

##### 2.2.3.5 Stress and burnout

Ten questions measured stress and burnout in relation to teacher’s work engagement in the school environment. The questions measured two aspects of teachers’ stress: *stress report* (*n* = 1), and *sources of stress* (*n* = 9). Teachers rated all items on a four-point scale ranging from 1 (Not at all) to 4 (A lot).

##### 2.2.3.6 Teachers’ motivation to leave

Teachers’ motivation to leave the profession was assessed using seven questions. Teachers rated all items on a four-point scale ranging from 1 (Not at all likely) to 4 (Very likely). Responses in all these domains were tested against the four core dimensions to check for relationships between them and how they might impact teachers’ well-being.

#### 2.2.4 Statistical analyses

All data analyses were performed using R version 4.3.1 (61). Descriptive analyses, including means, standard deviations, and intercorrelation coefficients, were presented for overall and individual core well-being dimensions, stress and burnout, and motivation to leave. Simple, multiple regression analysis and Analysis of variance (ANOVA) were used to examine the influence of demographic factors, stress and burnout, and motivation to leave on overall self-reported well-being dimensions. Additionally, the analysis assessed the collinearity among variables. Multivariate regression analysis was used to explore the influence of stress levels and motivation to leave the profession on each core teacher’s occupational well-being while adjusting for demographic variables as confounders.

### 2.3 Qualitative phase

#### 2.3.1 Design and data collection

This phase of the study addressed research question two and utilized a phenomenological qualitative research design to explore teachers’ perceptions of the impact of their professional experiences on their well-being and decision to remain in or leave the teaching profession? The phenomenological approach centers on understanding and interpreting individuals’ lived experiences within their natural contexts (62, 63). This approach is grounded in the philosophy that reality is constructed through individuals’ experiences and perceptions. By employing a phenomenological approach, the study delved deeply into teachers’ personal narratives and experiences, providing a rich, contextual understanding of how various factors influence their well-being within the educational setting (64). The choice of a phenomenological design was justified as it enabled an exploration of the lived experiences of teachers, facilitating a comprehensive understanding of the interplay between professional well-being, stress levels, teaching practices, and their motivation to leave the profession. This approach is well-suited to the research question, which aims to uncover the nuanced, deeply personal experiences of teachers that quantitative methods might overlook.

The last question on the survey was used to invite interested participants to the qualitative phase of the study. Recruitment of the interview participants was done through purposive sampling. Data were collected through semi-structured in-depth interviews, allowing for the flexibility to probe deeper into participants’ responses and explore emergent themes. The interview guide was developed and reviewed by external experts in educational psychology and qualitative research to ensure comprehensiveness and relevance. Interviews were conducted via phone call or video conferencing at various times based on the participants’ preferences and were recorded with their consent. Each interview lasted approximately 45–60 min, providing ample time to explore the participants’ experiences and perspectives thoroughly. Before the interview, participants were given a brief overview of the study’s purpose and the interview process, and their consent and demographic variables (context and years of teaching experience) were obtained. The interviews continued until data saturation was achieved and no new themes were identified (65). Participants were assured of the confidentiality of their responses. Interviews were transcribed verbatim, and all participants were offered the chance to review their transcripts to validate the accuracy and interpretation of their experiences.

#### 2.3.2 Qualitative data analysis

The transcribed data were coded and analyzed in NVivo (QSR International Pty Ltd.; Version 12 for Windows) by three of the authors (JCN, EA, and BMA) to ensure the credibility of the results. Data analysis was conducted using inductive thematic analysis, following Braun and Clarke’s six-phase framework, which is particularly suited to phenomenological studies (66). The analysis process involved familiarization with the data, generation of initial codes, searching for themes, reviewing themes, defining and naming themes, and producing the report. This approach allowed for the systematic organization and interpretation of the data, ensuring that the findings were grounded in the participants’ experiences. Throughout the analysis, reflexivity was maintained, with the researchers documenting their assumptions, biases, and reflections to ensure the credibility and authenticity of the findings (67). Peer debriefing and member checking were employed as additional checks to enhance the trustworthiness of the findings. Participants were entered into a draw to win one of four $50 gift cards to compensate the participants for their time and involvement in the study (64). To ensure anonymity, participants’ names were replaced with pseudonyms. Illustrative quotes were reported verbatim to support the study findings. The study was reported using COREQ guidelines (68).

### 2.4 Ethics approval

The James Cook University Human Research Ethics Committee granted ethics approval (H8638) for this study. Informed consent was obtained from all participants prior to their participation in the study. Participants had to cofirm their informed consent before proceeding with the survey. Verbal consent for participation in the interviews was obtained at the beginning of each session. To ensure privacy and confidentiality, all data were anonymized and securely stored.

## 3 Results

### 3.1 Quantitative findings

As shown in Table 1, the participants for the quantitative phase of the study were 247 Australian primary school teachers of whom 80% identified as females. Most of the participants (71%) worked in public schools, had Bachelor degrees (65%) and were aged between 30 and 39 (34%). Thirty percent (30%) of the participants had less than 1 year experience in teaching primary school children, followed by those who had 11–20 (24%) years of experience. Sixteen percent (16%) of them were involved in teaching grade 4 (16%) students, and the most represented state was New South Wales (30.4%). The majority of the teachers had received professional development training (88%).

**Table 1:** Participants’ profile.

Characteristic	*N* = 247
Gender, n (%)	
Female	195 (79)
Male	48 (19.4)
Other	4 (1.6)
Age, *n* (%)	
18–29	64 (26)
30–39	83 (34)
40–49	49 (20)
Above 50	46 (19)
Highest qualification qual, *n* (%)	
Bachelor’s degree	154 (65)
Master’s degree	64 (27)
PhD	7 (3.0)
Other	12 (5.1)
Teach experience, *n* (%)	
Less than 1	60 (25)
1–5	46 (19)
6–10	30 (13)
11–20	68 (29)
Above 20	33 (14)
Primary school experience, *n* (%)	
Less than 1	70 (30)
1–5	46 (19)
6–10	25 (11)
11–20	58 (24)
Above 20	38 (16)
School type, *n* (%)	
Other	5 (2.0)
Private	66 (27)
Public	173 (71)
Current class, *n* (%)	
Prep	28 (12)
Year 1	30 (13)
Year 2	25 (11)
Year 3	30 (13)
Year 4	36 (16)
Year 5	30 (13)
Year 6	27 (12)
Others	24 (10)
Class size, *n* (%)	
Less than 15	9 (3.9)
15–20	123 (53)
20–30	81 (35)
Above 30	18 (7.8)
Training, *n* (%)	
Yes	211 (88)
No	28 (12)

State		
Australian Capital Territory (ACT)	12	4.9
New South Wales (NSW)	75	30.4
Northern Territory (NT)	9	3.6
Queensland (QLD)	59	23.9
South Australia (SA)	21	8.5
Tasmania (TAS)	5	2
Victoria (VIC)	44	17.8
Western Australia (WA)	12	4.9

Table 2 presents the results of the association between the four dimensions of teacher occupational well-being (cognitive, subjective, physical & mental, social) and other factors such as stress, burnout and motivation to leave the profession. Cognitive well-being had significant positive correlations with social well-being (*r* = 0.403, *p* < 0.01) and subjective well-being (*r* = 0.325, *p* < 0.01). However, it had a negative correlation with physical/mental well-being (*r* = −0.170, *p* < 0.05). Subjective well-being showed significant negative correlations with physical/mental well-being (*r* = −0.526, *p* < 0.01) and stress and burnout (*r* = −0.490, *p* < 0.01), and a strong positive correlation with social well-being (*r* = 0.501, *p* < 0.01). Physical/mental well-being had a weak negative correlation with social well-being (*r* = −0.209, *p* < 0.01) but a significant positive correlation with stress and burnout (*r* = 0.517, *p* < 0.01). Social well-being had a weak negative non-significant correlation with stress and burnout (*r* = −0.126). Stress and burnout had a significant positive correlation with motivation to leave (*r* = 0.212, *p* < 0.01), indicating that stress is a predictor of motivation to leave. The internal consistency of each dimension was measured using Cronbach’s alpha, with values ranging from 0.60 to 0.90. The highest reliability was observed in physical/mental well-being (*α* = 0.90), while the lowest was seen in motivation to leave (*α* = 0.60). The mean scores across the dimensions ranged from 2.38 to 3.05, indicating moderate levels of occupational well-being among the teachers surveyed.

**Table 2:** Summary of the well-being dimensions and their inter-correlations, internal consistency and reliability.

Dimension	Number of items	1	2	3	4	5	6
Cognitive	12		0.325**	−0.170*	0.403**	−0.049	−0.055
Subjective	9			−0.526**	0.501**	−0.490**	0.141*
Physical and mental	10				−0.209**	0.517**	0.075
Social	15					−0.126	0.037
Stress and burnout	10						0.212**
Motivation to leave	7						
Mean ± SE		3.05 ± 0.51	2.89 ± 0.53	2.88 ± 0.91	3.05 ± 0.35	2.62 ± 0.04	2.38 ± 0.05
Cronbach’s alpha (95% CI)^a^		0.81 (0.77, 0.84)	0.81 (0.76, 0.85)	0.9 (0.88, 0.92)	0.86 (0.84, 0.88)	0.86 (0.82, 0.89)	0.6 (0.50, 0.67)

a 95% confidence interval.

***p < 0.001, **p < 0.01, *p < 0.05.

Table 3 presents the influence of teachers’ demographics (gender, age, experience, class size, and current teaching level) on their occupational well-being, stress levels, and motivation to leave the profession. Male teachers reported significantly lower physical/mental well-being (mean = 2.36) compared to female teachers (mean = 2.99, *p* < 0.001). There were no significant differences in cognitive, subjective, social well-being, or motivation to leave between male and female teachers. Teachers aged 50–60 and above 60 reported the highest cognitive well-being scores (mean = 3.13 and 3.37, respectively). Older teachers also showed the highest levels of subjective well-being (mean = 3.00 for the 50–60 age group and 3.31 for those above 60). However, physical/mental well-being was lower among the older age groups (mean = 2.64 for those above 60). Teachers aged 18–29 had the highest motivation to leave (mean = 2.52, *p* < 0.001), while older teachers (above 60) showed the lowest motivation to leave (mean = 1.90). Teachers with over 20 years of experience reported the highest level of cognitive well-being (mean = 3.31) and the lowest motivation to leave (mean = 1.98, *p* < 0.001). Those with 6–10 years of experience showed higher physical/mental well-being (mean = 3.25) but also higher stress and burnout levels (mean = 2.88, *p* < 0.01). Teachers with smaller class sizes (less than 15 students) reported lower motivation to leave (mean = 2.42) compared to those teaching larger classes (e.g., mean = 2.76 for classes of 30 students, *p* < 0.05). Class size had minimal influence on the other well-being dimensions. Prep and Year 5 teachers reported the highest cognitive well-being (mean = 3.16), while Year 4 teachers had the lowest level (mean = 2.90). Motivation to leave was significantly higher for teachers in Year 1 (mean = 2.63) and Year 2 (mean = 2.60), compared to those teaching higher grades (e.g., Year 6 mean = 2.38, *p* < 0.05). In summary, older and more experienced teachers reported higher cognitive and subjective well-being levels, while younger teachers had higher motivation to leave. Larger class sizes were associated with higher stress and motivation to leave.

**Table 3:** Impact of stress and burnout and motivation to leave on teacher occupational well-being dimensions.

Variables	Cognitive well-being	Subjective well-being	Physical/mental well-being	Social well-being	Stress and burnout (Sources of stress)	Motivation to leave
Demographic	Mean ± SE	Mean ± SE	Mean ± SE	Mean ± SE	Mean ± SE	Mean ± SE
Gender						
Female	3.06 ± 0.39	2.91 ± 0.41	2.99 ± 0.68	3.08 ± 0.25	2.64 ± 0.47	2.33 ± 0.40
Male	3.05 ± 0.78	2.93 ± 0.82	2.36 ± 0.14***	3.00 ± 0.95	2.45 ± 0.96	2.46 ± 0.76
Age						
18–29	2.95 ± 0.66	2.91 ± 0.54	2.90 ± 0.12	3.09 ± 0.52	2.56 ± 0.76	2.52 ± 0.61***
30–39	3.09 ± 0.58	2.84 ± 0.59	2.83 ± 0.12	3.03 ± 0.42	2.63 ± 0.73	2.45 ± 0.60***
40–49	3.05 ± 0.75	2.88 ± 0.86	2.87 ± 0.14	3.04 ± 0.40	2.64 ± 1.00	2.30 ± 0.74
50–60	3.13 ± 0.12	3.00 ± 0.13	2.89 ± 0.16	3.11 ± 0.64	2.60 ± 0.12	2.00 ± 0.85
Above 60	3.37 ± 0.90	3.31 ± 0.19	2.64 ± 0.31	3.15 ± 0.12	2.36 ± 0.20	1.90 ± 0.15
Experience in Primary school						
Less than 1	3.05 ± 0.67	2.90 ± 0.45	2.66 ± 0.11	3.09 ± 0.39	2.51 ± 0.71	2.61 ± 0.60***
1–5	3.14 ± 0.84	3.00 ± 0.98	2.84 ± 0.15	3.03 ± 0.66	2.38 ± 0.11	2.15 ± 0.78
6–10	3.04 ± 0.14	2.66 ± 0.16	3.25 ± 0.19	3.03 ± 0.67	2.88 ± 0.13**	2.33 ± 0.11
10–20	3.00 ± 0.62	2.87 ± 0.78	3.02 ± 0.11	3.06 ± 0.47	2.77 ± 0.80**	2.30 ± 0.73
20–30	3.31 ± 0.14	3.18 ± 0.19	3.23 ± 0.27	3.18 ± 0.14	2.89 ± 0.18**	1.98 ± 0.11
Above 30	3.00 ± 0.85	2.99 ± 0.94	2.67 ± 0.23	3.05 ± 0.99	2.50 ± 0.12	2.41 ± 0.10
Class Size						
Less than 15	2.86 ± 0.16	2.76 ± 0.12	3.16 ± 0.25	3.01 ± 0.16	2.42 ± 0.16	2.24 ± 0.19
15	2.99 ± 0.18	3.15 ± 0.15	2.28 ± 0.21	3.10 ± 0.14	1.88 ± 0.19	2.60 ± 0.19
20	2.98 ± 0.87	3.07 ± 0.64	2.75 ± 0.14	3.05 ± 0.51	2.38 ± 0.97	2.42 ± 0.70
25	3.15 ± 0.56	2.86 ± 0.69	2.99 ± 0.10	3.07 ± 0.39	2.69 ± 0.73*	2.34 ± 0.69
28	2.96 ± 0.84	2.86 ± 0.84	2.88 ± 0.15	3.07 ± 0.52	2.80 ± 0.95*	2.39 ± 0.81
30	3.16 ± 0.75	2.81 ± 1.00	2.75 ± 0.19	3.05 ± 0.62	2.66 ± 0.85*	2.40 ± 0.90
Other	3.17 ± 0.19	3.22 ± 0.99	3.02 ± 0.26	3.28 ± 0.90	2.76 ± 0.20	2.20 ± 0.29
Current Class						
Prep	3.16 ± 0.91	2.98 ± 0.78	3.12 ± 0.15	3.01 ± 0.16	2.55 ± 0.10	2.44 ± 0.12*
Year 1	3.01 ± 0.11	2.99 ± 0.12	2.90 ± 0.19	3.10 ± 0.14	2.58 ± 0.14	2.63 ± 0.93*
Year 2	3.10 ± 0.91	2.84 ± 0.76	3.02 ± 0.19	3.05 ± 0.05	2.60 ± 0.11	2.51 ± 0.93
Year 3	3.13 ± 0.84	3.00 ± 0.96	2.74 ± 0.15	3.12 ± 0.50	2.58 ± 0.10	2.37 ± 0.88
Year 4	2.90 ± 0.10	2.81 ± 0.13	2.60 ± 0.17	2.94 ± 0.71	2.63 ± 0.12	2.39 ± 0.91
Year 5	3.17 ± 0.10	2.86 ± 0.11	2.86 ± 0.20	2.99 ± 0.84	2.51 ± 0.12	2.24 ± 0.13
Year 6	3.00 ± 0.11	2.84 ± 0.11	2.90 ± 0.24	3.03 ± 0.71	2.63 ± 0.16	2.38 ± 0.10
Other	2.97 ± 0.11	2.94 ± 0.12	2.77 ± 0.17	3.14 ± 0.71	2.72 ± 0.14	2.00 ± 0.81

95% Confidence Interval for Mean. ***p < 0.001, **p < 0.01, *p < 0.05.

Table 4 presents the results of the regression analyses examining the relationships between various demographic and work-related variables with the four dimensions of well-being: cognitive, subjective, physical/mental, and social. Male teachers reported significantly lower physical and mental well-being than female teachers (beta = −0.28, *p* = 0.031), indicating that gender plays a role in this aspect of well-being. However, there were no significant gender differences in cognitive, subjective, or social well-being. The coefficients for age-related, school type, qualification, and class size comparisons across all well-being dimensions were not statistically significant. However, teachers who had undergone training reported significantly higher social well-being levels (beta = 0.24, *p* = 0.013) and marginally higher subjective well-being (beta = 0.22, *p* = 0.065). Additionally, training was associated with lower physical and mental well-being (beta = −0.32, *p* = 0.073). Teaching Years 1, 3 and 4 was strongly associated with higher physical and mental well-being (Year 1 beta = 0.51, *p* = 0.022; Year 3 beta = 0.63, *p* = 0.003; Year 4 beta = 0.45, *p* = 0.031). Experiencing stress at work significantly impacted physical and mental well-being (beta = 0.50, *p* < 0.0001), while addressing parent or guardian concerns negatively impacted physical and mental well-being (beta = −0.23, *p* = 0.003) but positively impacted social well-being (beta = 0.14, *p* = 0.001). Being intimidated or verbally abused by students was also associated with a decline in physical and mental well-being (beta = 0.16, *p* = 0.016). Among the variables related to motivation to leave, only “Taking a break from work” was significantly negatively associated with subjective well-being (beta = −0.14, *p* = 0.004). The models for subjective and physical/mental well-being had higher explanatory power, with adjusted R-squared values of 0.47 (*p* < 0.001) and 0.61 (*p* < 0.001), respectively. The cognitive and social well-being models had lower explanatory power, indicating that other unmeasured factors may be influencing these dimensions.

**Table 4:** Factors influencing the four core well-being dimensions.

Variables	Cognitive well-being			Subjective well-being			Physical and mental health well-being			Social well-being		
	beta	SE	*p*-value	beta	SE	*p*-value	beta	SE	p-value	beta	SE	*p*-value
Demographic												
Male (Ref: Female)	−0.01	0.11	0.963	−0.11	0.09	0.233	**−0.28**	**0.13**	**0.031**	−0.12	0.07	0.095
Age (Ref: 18–29 years)												
30–39	0.07	0.12	0.539	−0.11	0.10	0.279	0.13	0.14	0.361	−0.08	0.08	0.318
40–49	0.01	0.13	0.965	−0.03	0.11	0.812	0.20	0.16	0.216	−0.07	0.08	0.433
Above 50	0.06	0.14	0.702	−0.03	0.12	0.769	0.12	0.17	0.501	0.00	0.09	0.967
School type (Ref: Other)												
Private	−0.17	0.35	0.632	−0.36	0.29	0.214	0.70	0.42	0.101	−0.04	0.23	0.868
Public	−0.20	0.35	0.565	−0.34	0.28	0.235	0.60	0.41	0.148	−0.02	0.22	0.921
Training (No)												
Yes	0.04	0.15	0.768	0.22	0.12	0.065	−0.32	0.18	0.073	**0.24**	**0.09**	**0.013**
Highest qualification (Ref: Bachelor)												
Master’s degree	−0.04	0.10	0.663	−0.02	0.08	0.848	0.05	0.12	0.651	−0.05	0.06	0.413
PhD	0.26	0.26	0.328	0.08	0.21	0.708	−0.43	0.31	0.170	−0.16	0.17	0.329
Other	0.11	0.22	0.611	0.25	0.18	0.152	−0.18	0.26	0.489	0.07	0.14	0.625
Class size (<15)												
15–20	0.17	0.22	0.445	−0.05	0.18	0.764	0.24	0.26	0.360	0.08	0.14	0.571
20–30	0.20	0.23	0.391	−0.11	0.19	0.576	0.26	0.27	0.352	0.09	0.15	0.548
Above 30	−0.06	0.27	0.819	−0.22	0.22	0.308	0.37	0.32	0.241	0.04	0.17	0.820
Current class (Ref: Prep)												
Year 1	0.19	0.18	0.313	0.04	0.15	0.772	**0.51**	**0.22**	**0.022**	0.01	0.12	0.937
Year 2	0.28	0.18	0.131	0.24	0.15	0.118	0.07	0.22	0.742	0.19	0.12	0.109
Year 3	0.29	0.18	0.101	0.04	0.14	0.787	**0.63**	**0.21**	**0.003**	0.08	0.11	0.451
Year 4	0.33	0.17	0.058	0.16	0.14	0.273	**0.45**	**0.21**	**0.031**	0.01	0.11	0.898
Year 5	0.14	0.17	0.410	0.00	0.14	0.981	−0.23	0.20	0.253	−0.05	0.11	0.670
Year 6	0.30	0.18	0.094	−0.01	0.15	0.956	0.21	0.21	0.334	0.01	0.11	0.961
Others	0.21	0.18	0.242	0.11	0.14	0.452	0.23	0.21	0.268	0.14	0.11	0.225
Motivation to leave												
To get a degree in education	0.05	0.05	0.405	0.06	0.04	0.169	−0.08	0.07	0.205	0.02	0.03	0.502
Take a job outside of education	−0.06	0.06	0.354	−0.08	0.05	0.098	−0.03	0.07	0.687	−0.05	0.04	0.227
Attend to family responsibility	0.00	0.07	0.980	0.05	0.06	0.376	0.08	0.08	0.333	0.03	0.04	0.545
Take a break from work	0.08	0.06	0.181	**−0.14**	**0.05**	**0.004**	0.04	0.07	0.525	−0.04	0.04	0.252
reach retirement	−0.03	0.06	0.668	−0.01	0.05	0.866	0.04	0.07	0.552	−0.01	0.04	0.737
Feel left out of things	−0.01	0.05	0.922	−0.05	0.04	0.309	0.02	0.06	0.737	0.02	0.03	0.472
Feeling awkward and out of place	0.00	0.03	0.995	0.01	0.03	0.647	0.02	0.04	0.703	0.02	0.02	0.381
Stress and burnout												
I experience stress in my work	0.06	0.07	0.346	−0.03	0.05	0.553	**0.50**	**0.08**	**<0.0001**	−0.07	0.04	0.083
Having too little time for lesson preparation	−0.04	0.07	0.605	−0.11	0.06	0.065	0.13	0.09	0.154	0.07	0.05	0.163
Maintaining classroom discipline	−0.09	0.06	0.110	−0.03	0.05	0.539	0.07	0.07	0.317	−0.03	0.04	0.451
Having too many lessons to teach	−0.05	0.06	0.442	−0.05	0.05	0.298	−0.01	0.08	0.919	−0.04	0.04	0.318
Modifying lessons for students with special needs	0.05	0.06	0.421	0.06	0.05	0.209	0.04	0.07	0.611	0.04	0.04	0.343
Being intimidated or verbally abused by students	−0.06	0.06	0.309	0.01	0.05	0.811	**0.16**	**0.07**	**0.016**	−0.03	0.04	0.377
Having too much administrative work to do	0.05	0.08	0.496	−0.06	0.06	0.364	−0.09	0.09	0.298	−0.02	0.05	0.723
Having too much <marking>	−0.10	0.07	0.157	−0.07	0.06	0.240	0.10	0.08	0.253	−0.06	0.04	0.158
Addressing parent or guardian concerns	**0.18**	**0.07**	**0.007**	0.06	0.05	0.247	**−0.23**	**0.08**	**0.003**	**0.14**	**0.04**	**0.001**
Being held responsible for students’ achievement	−0.06	0.06	0.332	−0.01	0.05	0.899	0.06	0.07	0.372	−0.03	0.04	0.419
Model summary statistics	Adjusted-R^2^ = 0.21, *F =* 0.97, *p* > 0.05			Adjusted-R^2^ = 0.47, *F =* 3.28, *p* < 0.001			Adjusted-R^2^ = 0.61, *F =* 5.84, *p* < 0.001			Adjusted-R^2^ = 0.28, *F =* 1.49, *p* < 0.05		

***p < 0.001, **p < 0.01, *p < 0.05. R^2^ = Coefficient of determination, SE = Standard error, beta = Regression coefficient, F = F-statistic.

### 3.2 Qualitative findings

#### 3.2.1 Profile of participants in the qualitative phase

A total of 21 Australian primary school teachers comprising, classroom teachers (*n* = 11), specialist teachers (*n* = 4), learning support teachers (*n* = 2), and school leaders (*n* = 4) participated in this phase of the study. Most of the teachers were female (95%) and worked in public schools (59%). The participants were from five Australian states - Australian Capital Territory (9.5%), New South Wales (24%), Northern Territory (19%), Queensland (24%) and Tasmania (24%). The teachers were aged between 29 to 62 years and had between 1 and above 20 years of teaching experience. Class size ranged from 7 to >30. Most of the teachers had a Bachelor degree (57%). Ninety-five percent of the teachers had participated in in-service training (95%) and some of them teach composite classes (28.6%). Composite classes are classes that have more than one grade level (e.g., Years 2/3).

### 3.3 Emerging themes

Five themes were identified in this study. The key themes identified are (1) job satisfaction and motivation, (2) poor work overload, (3) leadership support, (4) poor work-life balance, and (5) professional relationships and collegial support. Teachers’ decisions to remain in or leave the profession are influenced by multiple factors. While many teachers enter the profession with high motivation and passion, excessive workload, stress, lack of support from leadership, and poor work-life balance contribute to job dissatisfaction and burnout. Positive relationships with colleagues and strong leadership can provide support, but these are often insufficient to counteract the negative impacts.

#### 3.3.1 Theme 1: job satisfaction and motivation

Initially, many teachers entered the profession with enthusiasm and passion for teaching. Most teachers reported that they love their job and get job satisfaction from teaching children and working with colleagues. Teachers love the fact that they interact with children and build a rapport with them. It gives them satisfaction to help the children reach their goals. Aside from teaching, teachers also help children with their emotional needs while in the classroom which brings joy and gives satisfaction to teachers knowing that they have helped troubled children.

*“So as a primary school teacher, I have a lot of satisfaction My class was amazing, you know, I really loved being at work. I loved my job. I loved everything about it. It filled my bucket. There were stressful times, you know. I had to write reports and do all those sorts of things, but never enough to be an issue. I’m quite happy with how things are going at the moment. I have good job satisfaction at the moment. Job satisfaction for me includes good collegial relationships. We’re working collaboratively. So, we have a bit of fun sometimes. So, we have got good relationships with each other” (Penny).*

*“As far as the kids go, most of the time I enjoy the classes that I had, even though there could be several difficult kids but over time you get to know them and so that makes it easier. (Lally).*

However, over time, this motivation often wanes due to increasing demands and lack of support. The teachers reflected on the change in their feelings toward teaching and described the significant drop in motivation over time.

*“It declined rapidly from 2020 onwards. So, not very happy at all really. It just does not satisfy me. If you have got a really good team and your colleagues and you work effectively as a team, and you are well supported by leadership. Then it’s actually good but if that is not in place, then no.” Towards the end of last year my motivation was like a zero. I just did not feel satisfied with what I was doing so I did not want to keep doing it. But before then, probably around 7 or 8.” (Wet).*

*“At first, I was happy because I have passion for teaching, but as I progressed, I saw how the demand was just too much. You know, the demand was just too much…” (Zem). “You do feel stretched. And you do feel like you could do more if you had more time, or you know things like that. You do see little improvement; you do fill little gaps. Yeah, it does make a difference. The only thing is, you feel you could make more of a difference if you have more resources or more time, or things like that” (Raba).*

*“I loved going to work even if I’d have had awful days, you know, children throwing things and more stuff going on but I always woke up in the morning looking forward to going to work, whereas now I do not know whether it’s just 6 years in or because of the school I’m at or the students that I’m teaching. I dread, going to work most days now, I just do not want to do it.” (Esan).*

Interestingly, some teachers expressed their strong commitment to their students due to their passion for the job, even when overall motivation and job satisfaction declined. Despite the initial enthusiasm, many teachers reported becoming disillusioned over time due to various challenges in the profession.

*“And it was worth coming back to it. So, yes, I absolutely love my job in that sense (But) I have a limit on how much I can get done. Because teaching I think… our to-do list never ends. It’s basic and ongoing. So that can decrease my motivation, I suppose. I want to do as best as I can, that’s for sure.…. [However],* if I had my time again, I never would have become a teacher. It is, for the most part, heads down and one of the most dissatisfying things I’ve ever done. And I only do it now for the money until I finish my current studies when I can leave.” *(Giks).*

#### 3.3.2 Theme 2: work overload

Teachers frequently cited overwhelming workloads and resultant stress as significant factors affecting their well-being and job satisfaction. Many described the crushing weight of additional hours and tasks, which often extend beyond the school day and into weekends.

*“I think, in general, workload has the greatest impact on my well-being. The workload over 30 years, you would think that you know, an experienced teacher would potentially not have to work as hard because you have finetuned all your teaching skills. It’s just, workload in general I feel has increased, and impacted my well-being and the well-being of my colleagues. (Sod).*

*“It’s absolutely like its crushing workload. So, I’m not satisfied with the extra hours I have to put in. 10 h a week extra minimum. Minimum, yeah, because when the report writing comes on twice a year, that will go up to about 15 h. It’s not very sustainable to continue with this workload. (Mira).*

#### 3.3.3 Theme 3: poor work-life balance

Teachers reported that the demands of their jobs impact on their personal lives, making it challenging to maintain a healthy work-life balance. Many reported that the excessive workload led to stress, exhaustion, and burnout, negatively impacting their social relationships and personal time.

*“I do believe. It’s impacting my occupational well-being. I suppose, I’m probably more tired, and more stressed, and more mentally drained than I’ve ever been in teaching, and it makes me, I mean just like anything, if you are tired, exhausted and drained, you are not a great person. Like you will not do things as well as you can. So, I sometimes think it does affect the way I teach. I’m probably not as patient as I used to be. Yeah, I probably had more sick days off as well. You are probably looking at more colds and flu and things because I’m run down a little bit, but because no one else wants to teach these kids and I do. I want to make a difference so that’s why I do what I do, but it does affect my occupational well-being definitely” (Val).*

*“A thousand percent. Start at 7:00 in the morning and return home by 4: 30. (Plus) at least 2 h every night. Weekends are full on. On weekends, at least 8 or 9 h, because I’m a contract teacher covering somebody. I’ve got 14–16 h days on the weekend getting things done. It’s like there is really no time to go to anything else.” (Tak).*

They shared how the constant work demands affected their health. They also noted the difficulty of balancing teaching with personal responsibilities. The stress associated with these excessive demands takes a toll on their mental and physical health.

*“I do not sleep very well. Mine is a headache. I have more frequent headache. As at last year; it was almost daily. But as of this year, not as often as last year.” (Zem).*

*“The adding on, the piles of work, and the stress, and all of that. It just affects your sleep at times. My mood while I’m working. There’s always feeling on edge and the adrenaline of having no time to do anything and running around too much like; cause it’s too much.” (Mira).*

#### 3.3.4 Theme 4: impact of leadership support

The participants reported that the quality of leadership and support within schools is a critical determinant of teachers’ job satisfaction and their decision to stay or leave. Teachers who felt unsupported by their school leadership often reported higher levels of stress and lower motivation. Systemic issues and a perceived lack of appreciation for teachers’ efforts contributed to their dissatisfaction. They also highlighted the pivotal role of leadership, emphasizing the demotivating effect of the absence of support.

*“Unfortunately, it should not be like that, and it does not have to be like that, but with my experience, it literally just comes down to leadership. When you are stressed, and working in a toxic environment where you know it’s just a broken system because staff well-being is not on the agenda, and staff well-being is not thought of. It’s very hard to stay motivated and in that environment, my motivation is very low.” (Yabby).*

*“If you have got a really good team and your colleagues and you work effectively as a team, and you are well supported by leadership. Then it’s actually good but if that is not in place, then no.” (Wet).*

Participants recounted their ordeal and highlighted how unsupportive environments exacerbated burnout.

*“It’s complicated. I taught for a few years in Queensland. And by then I was completely, utterly, burnt out. I was burnt out, I had to leave and for a long time, I would not even contemplate returning to a classroom. As I said I’ve had experiences. Not good schools, not good executives, no support at all.”*

#### 3.3.5 Theme 5: professional relationships and collegial support

The participants stated that positive relationships with colleagues and a supportive work environment can mitigate some of the stress and contribute to job satisfaction. Many teachers value the camaraderie and peer support they receive from their colleagues. They highlighted the importance of good relationships with colleagues, particularly the understanding and support among older, experienced teachers.

*“We support each other. We provide each other with peer support. We do lots of crying with each other when there is so much stress. We can do phone calls to support each other too. (Yaby).*

*“Pretty good professional work relationship. They are pretty understanding. Most of us are older teachers too. We are already in our forties. You know, it’s not like we are 20 years old. Most of us are dinosaurs by now. I do have a good professional relationship with them. Yeah, we are all teachers, and we all need help because it is hard work, teaching is lovely, it’s fun but it’s a lot” (Val).*

Triangulation of the quantitative and qualitative findings is presented in Table 5. Generally, the teachers described their occupational well-being in varying ways that brought to the fore, the deep-seated issues many teachers face, ranging from initial enthusiasm to eventual disillusionment due to the heavy toll of workload and systemic challenges on their health and motivation (see Table 5).

**Table 5:** Triangulation of study findings.

Identified themes	Triangulation of findings
Job satisfaction and motivation	Quantitative data indicates that motivation to take a break was associated with lower subjective well-being, suggesting that work-related stress and exhaustion may contribute to decreased well-being (beta = −0.14, p = 0.004). This aligns with the qualitative theme of declining job satisfaction and motivation. Teachers entered the profession with high levels of passion, but over time, many reported a drop in motivation due to increasing work demands and a lack of support. The qualitative accounts of teachers feeling stretched and unable to adequately fulfill their role due to limited resources and time constraints confirm the quantitative results, where the motivation to leave and dissatisfaction were tied to burnout and excessive work demands.
Work overload	Both the qualitative and quantitative data strongly highlight the impact of work overload on teachers’ well-being. In the quantitative findings, stress and burnout were significantly associated with reduced physical and mental well-being (beta = 0.50, *p* < 0.001). Qualitative findings confirmed this, where teachers expressed that excessive workload, such as lesson preparation, after-hours work, and administrative tasks, negatively impacted their mental health and contributed to stress. Teachers shared how heavy workload impacted their personal time and weekends, underscoring the overwhelming nature of their responsibilities.
Work-life balance	Quantitative results showed a significant relationship between stress from work and diminished physical and mental well-being (beta = 0.50, *p* < 0.001). This strongly correlates with the qualitative theme, where teachers reported how work demands encroached on their personal lives, affecting their health, relationships, and overall quality of life.
Leadership support	The qualitative findings emphasized the critical role of leadership in influencing teachers’ occupational well-being. Teachers who felt unsupported by school leadership often reported higher levels of stress and a lack of motivation. This is supported by the quantitative results, where teachers described how toxic environments and poor leadership contributed to burnout.
Professional relationships and collegial support	Quantitative results suggested that social well-being was positively associated with collegial support and leadership (beta = 0.24, *p* = 0.013). This confirms the qualitative findings where teachers emphasized the importance of collegial relationships in mitigating stress. Teachers reported that supportive relationships with colleagues helped them navigate stressful work environments. However, the quantitative data also suggest that while social support is beneficial, it is often insufficient to fully counteract the negative effects of workload and poor leadership.

## 4 Discussion

The findings from this sequential explanatory mixed-methods study shed light on the complex relationships between teacher occupational well-being, teaching engagement, stress, and motivation to either stay in or leave the profession. This discussion integrates the quantitative and qualitative results, offering a triangulated perspective on the key drivers behind teacher occupational well-being, stress, and retention, while also providing insights into possible strategies to improve teacher satisfaction and reduce attrition.

This study indicates that high self-efficacy significantly contributes to job satisfaction and positively impacts teacher occupational well-being. Teachers in the study consistently reported confidence in their ability to engage students and manage classroom behavior effectively. This aligns with previous research highlighting the role of self-efficacy in job satisfaction and teacher occupational well-being (69, 70). The quantitative data showed that high self-efficacy reduced stress and psychosomatic symptoms, while the qualitative findings revealed that teachers derive satisfaction from helping students reach their goals. These findings are consistent with earlier work by Caprara et al. (71) and Ortan et al. (72), who found that high self-efficacy contributes to job satisfaction and well-being.

However, the qualitative data also highlights a paradox: while teachers feel competent in their instructional roles, many are overwhelmed by excessive workloads, which diminish their overall occupational well-being. Teachers reported that excessive planning, grading, and administrative tasks encroach on personal time, leading to stress and burnout. These accounts are supported by the quantitative data showing that increased workload leads to heightened stress levels and negatively impacts physical and mental well-being, echoing findings from Lawrence et al. (73) on the detrimental effects of non-teaching-related tasks. Additionally, the study highlights the substantial toll that stress and burnout take on teachers’ health. The quantitative data shows a clear association between stress and burnout and increased psychosomatic symptoms. Teachers in the qualitative phase corroborated this, describing a range of physical and mental health issues, including headaches, fatigue, and insomnia, as a direct result of their workload and stress. This is consistent with previous studies showing that job demands are highly associated with fatigue and psychosomatic complaints (74, 75). The teachers’ accounts of how the profession negatively affected their health reflect the broader trend of teachers struggling to maintain a work-life balance amidst overwhelming job demands. While social support from colleagues and leadership helped to alleviate some stress, it was often insufficient to fully counteract the negative health impacts of teaching. The study’s findings echo earlier research by Liu and Ramsey (76) and Simbula (77), which emphasized the harmful effects of high job demands and stress on teacher health and well-being.

Building on these findings, the study suggests that younger, less experienced teachers are more likely to consider leaving the profession due to stress and burnout. The quantitative data show that teachers aged 18–39 are more motivated to leave compared to their older counterparts, a trend supported by the qualitative findings where early-career teachers like Zem, reported feeling overwhelmed by the demands of the profession. This aligns with previous research indicating that early-career teachers are at higher risk of burnout and attrition (29, 78). The reason for being overwhelmed might stem from the fact that younger teachers often hold high self-efficacy beliefs but lower outcome expectancies which usually leads to frustration and intention to leave the profession (79). In contrast, more experienced teachers reported higher stress levels but were less inclined to leave the profession, possibly due to greater resilience or a deeper commitment to their students. However, workload remained a key concern for both experienced and early-career teachers. As several teachers in the qualitative phase explained, they felt constantly overwhelmed by the volume of work, contributing to long hours and a sense of unsustainable pressure, these findings are consistent with the work of Bermejo-Toro et al. (27) and Walter and Fox (28).

Furthermore, both the qualitative and quantitative findings highlight the negative impact of large class sizes on teacher occupational well-being. The quantitative data showed that teachers with larger class sizes experienced higher stress levels, a finding supported by teachers’ qualitative reflections on the challenges of managing large groups of students while balancing administrative tasks and lesson planning. These findings are consistent with prior research by Fuenzalida (5), which identified class size as a significant factor influencing teacher occupational well-being.

Despite the challenges, many teachers found fulfillment in their interactions with students, particularly in helping them achieve milestones. This relationship with students was a source of motivation and job satisfaction, as noted in previous research by Ortan et al. (72) and Hilger et al. (80). The teachers emphasized the joy they derived from building strong connections with their students, even in the face of broader systemic challenges. Additionally, the study underscores the pivotal role of social support in mitigating stress and enhancing teacher occupational well-being. Teachers who received strong support from colleagues and school leadership reported higher job satisfaction and lower burnout levels. The teachers described how collegial support and positive relationships with leadership helped them navigate the pressures of their roles. This aligns with previous research emphasizing the critical role of social support in enhancing teacher occupational well-being (47, 81). Conversely, a lack of leadership support was frequently cited as a source of dissatisfaction and stress, contributing to teachers’ intentions to leave the profession. One teacher’s account of working in a toxic environment, where staff well-being was neglected, illustrates how poor leadership exacerbates burnout. These findings mirror those of Skinner et al. (82), who found that inadequate support from leadership increases stress levels, further confirming the importance of supportive school environments in retaining teachers (19).

### 4.1 Practical implications and recommendations

The practical implications of this mixed-methods study highlight several key recommendations for improving teacher occupational well-being, including addressing self-efficacy, leadership support, workload, and professional development. These findings offer actionable insights for educators, school leaders, and policymakers.

#### 4.1.1 Enhancing self-efficacy through professional development

The study highlights the critical role of self-efficacy in influencing teacher occupational well-being, job satisfaction, and classroom management. Teachers with high self-efficacy reported better relationships with students and stakeholders, which led to increased job satisfaction and improved well-being (83, 84). Aligned with Bandura’s social learning theory (85), individuals often learn teaching methods by observing and modelling experienced teachers, a practice embedded in teacher education programs for pre-service teachers. However, novice teachers with full-time duties frequently lack ongoing opportunities to observe and model, potentially stunting their professional growth (79). Bandura posits that individuals with high self-efficacy set ambitious goals and are dedicated to achieving them (86). Applying this to novice teachers, those with strong self-efficacy are more likely to aspire to excellence, set high classroom goals, and commit deeply to their teaching roles. Research indicates that support from more experienced teachers in the form of induction programs that enhance self-efficacy are crucial, during the early stages of a novice teacher’s career, as they not only ease the transition from university to classroom but also bolster commitment to teaching quality and professional development (87). Engaging young teachers in programs that boost self-efficacy from the start is essential, as their belief in their own effectiveness is likely influenced by their initial experiences and successes with new teaching strategies (88). Research has shown that teachers exposed to regular professional development report higher job satisfaction (89). Targeted training programs that address immediate classroom challenges can also help alleviate stress and boost teacher confidence.

#### 4.1.2 Supporting teacher occupational well-being through leadership

The study found that leadership support is crucial for teacher occupational well-being. Teachers who lacked support from school leaders experienced lower self-efficacy and job satisfaction, emphasizing the need for strong, proactive leadership (90–92). School administrators should focus on building a supportive, inclusive work environment by offering recognition, emotional support, and regular bottom-up communication. Leadership training programs should also prioritize staff well-being, equipping leaders to address teacher concerns effectively and provide guidance, particularly to early-career teachers.

#### 4.1.3 Managing workload and reducing stress

Excessive workload was a significant source of stress and burnout in this study, which negatively impacted teacher health. Teachers described their workload as overwhelming and unsustainable. Policies should be implemented to reduce non-teaching duties, streamline administrative tasks, and reassess the necessity of certain responsibilities (34, 93). Schools could hire additional administrative staff to handle paperwork and allow teachers to focus on instruction, which would alleviate some of the emotional exhaustion associated with heavy workloads.

#### 4.1.4 Promoting social support and collaboration

Social support from colleagues and leadership was shown to mitigate stress and improve teacher occupational well-being. Teachers who had strong collegial relationships reported higher job satisfaction and lower stress levels (47, 81, 94). Schools should foster collaboration by promoting team teaching, peer mentoring, and informal professional networks. Providing opportunities for teachers to collaborate and offer mutual support can reduce individual workloads and strengthen professional relationships (93). Social gatherings and peer support groups can also enhance collegial bonds and improve the overall work environment.

#### 4.1.5 Addressing class size and resource allocation

Large class sizes were linked to increased stress and burnout, particularly for teachers in junior grades. Policymakers and school administrators should work to reduce class sizes or ensure that teachers have adequate resources, such as teaching assistants or technology, to manage large classes more effectively (5). This would help alleviate the stress associated with managing diverse student needs, particularly in differentiated classrooms.

#### 4.1.6 Supporting work-life balance

Work-life balance emerged as a recurring theme, with many teachers struggling to set boundaries between work and personal life. Schools should actively promote work-life balance by encouraging teachers to set limits on after-hours work and reduce the pressure to work outside of school hours. Policies that limit email communication after work, offer flexible working arrangements, or provide wellness programs—such as mindfulness sessions or access to mental health resources—can help teachers manage stress and prevent burnout.

#### 4.1.7 Supporting early-career teachers

Early-career teachers were found to be particularly vulnerable to stress and burnout. Schools should develop structured mentorship programs to support new teachers in managing their workload, improving classroom management, and building resilience. Providing early-career teachers with regular feedback, professional development, and emotional support from experienced colleagues can improve their job satisfaction and reduce the likelihood of attrition (29, 95, 96).

#### 4.1.8 Leveraging teacher-student relationships

Positive teacher-student relationships were identified as a key factor in teacher motivation and job satisfaction. Schools should prioritize strategies that allow teachers to spend more quality time building rapport with students, as these relationships foster a positive learning environment and enhance teacher occupational well-being (28, 93). Professional development programs focused on relationship-building and classroom management can help teachers engage more effectively with students, improving both student outcomes and teacher satisfaction.

#### 4.1.9 Improving teacher health and mental health support

The study found that teaching negatively impacted both the physical and mental health of teachers. Schools should offer accessible mental health support services, such as counseling and stress management workshops, and ensure that teachers have access to mental health days or flexible leave policies. Partnerships with external organizations could provide workshops on coping strategies for stress and anxiety, which would support teachers’ long-term health and well-being (77, 97).

#### 4.1.10 Recognizing and supporting experienced teachers

Experienced teachers showed higher stress levels but were less likely to leave the profession compared to their early-career counterparts. Schools should recognize the contributions of experienced teachers by involving them in decision-making, leadership roles, or mentorship programs. Offering opportunities for career advancement and reducing workload in later stages of their career can help retain experienced teachers and improve their well-being (34, 93).

### 4.2 Future research

Considering the extensive findings discussed in this study, future research should consider the evolving challenging nature of the teaching profession. Understanding the workload experiences and stressors associated with the teaching profession can inform tailored support structures and professional development opportunities. This study was dominated by females, future studies should include participants across all gender identities to promote increased diversity in research findings. Additionally, there is a need to also investigate the role of school leadership in ensuring early career teachers and experienced teachers receive support targeted to their individual needs for positive well-being that enables teacher retention. By continuing to explore and address the factors impacting teacher occupational well-being, stakeholders can work towards creating a more supportive and healthy school environment for all stakeholders.

### 4.3 Strengths and limitations

This study is not without limitations, while the study provides valuable insights into teacher occupational well-being, certain limitations should be considered when interpreting its findings. The study focuses specifically on Australian primary school teachers, which may restrict the generalizability of the results to individuals in other occupations and may not be applicable across all educational contexts or regions and our sample may not be representative of all Australian teachers. Additionally, the data analyzed was derived entirely from self-reported questionnaires and interviews, which increased the likelihood of response bias. Furthermore, the study sample has more female participants than males which may further limit the study’s broader applicability.

Nevertheless, the strength of this study is its use of mixed methodology, which allows for complete and synergistic utilization of data to validate findings from qualitative and quantitative data sources that only one of the methods could not have done. This approach enhanced the comprehensiveness of the findings. Additionally, the holistic exploration of the relationship between the factors considered and teacher occupational well-being is important for policy makers, administrators, and educators aiming to create more supportive and effective educational environments.

## 5 Conclusion

The relationship between teachers’ well-being in the workplace, professional engagement, stress level, and motivation to leave or stay in the profession was investigated using the OECD occupational well-being framework. The study’s findings confirm the complex interplay between self-efficacy, workload, social support, stress, and teacher occupational well-being. Teacher self-efficacy and job satisfaction were significantly influenced by social support structures and positive well-being policies enacted by school leadership that enabled effective classroom management and good relationship-building with children. While high self-efficacy and social support can buffer the negative effects of stress and contribute to job satisfaction, excessive workloads, and inadequate leadership support lead to burnout and attrition. Stress-related psychosomatic symptoms as a result of an unbearable and unsustainable workload impacted the health and well-being of teachers leading to a desire to leave the profession. Addressing these issues through better workload management, stronger leadership, and increased support for teachers is critical for sustaining teacher occupational well-being and reducing turnover. Further research and policy initiatives should focus on creating more sustainable working conditions to retain both early-career and experienced teachers in the profession.

## Data Availability

The original contributions presented in the study are included in the article/Supplementary material, further inquiries can be directed to the corresponding author.
