# Supplementary material for: From passion to pressure: exploring the realities of the teaching profession
Source: Front Public Health. 2025 Mar 7;13:1505330. doi: 10.3389/fpubh.2025.1505330 (PMC11925896; doi:10.3389/fpubh.2025.1505330)
Supplement: Supplementary file 1 [file Data_Sheet_1.pdf]

Appendices

Appendix 1

PISA 2021 TQ Indicators Questionnaire

Please provide responses that best represent your opinion about each of these statements.

Please attempt to answer all questions.

**School and Teacher Characteristics**		
**Question**	**Answer scale**	
Are you a registered mainstream primary classroom teacher?	○ Yes<br>○ No	
What is your age group?	18-29 30-39 40-49 50-60 >60	
What is your gender?	Male	Female<br>Other
Are you a public or private primary school mainstream teacher?	Public	Private
Have you received any training or taken part in any professional development courses since you started teaching?	Yes	No
If yes, what type of training was it (specific)?		
What is your highest qualification?		
How many years have you been teaching?		
How many years have you been teaching in mainstream primary classroom?		
What state do you currently work in?		
What class do you currently teach?	Prep, Years 1, 2, 3, 4, 5, 6, Other ____please specify	
How many children are in your class?		
What types of learning needs do you encounter in your class?		

**Indicators**	**Questions**	**Items**	**Answer scale**
**Stress and burnout**			1-Not at all<br>2- To some extent<br>3- Quite a bit<br>4- A lot
**Stress report**	In your experience as a teacher at your school, to what extent do the	I experience stress in my work	

	following occur?		
**Sources of stress**	Thinking about your job at your school, to what extent are the following sources of stress in your work?	Having too little time for lesson preparation	
		Maintaining classroom discipline	
		Having too many lessons to teach	
		Modifying lessons for students with special needs	
		Being intimidated or verbally abused by students	
		Having too much administrative work to do	
		Having too much <marking>	
		Addressing parent or guardian concerns	
		Being held responsible for students' achievement	
**Motivation to leave teaching**			
	How likely are each of the following factors to be a cause for you to leave classroom teaching?	To be promoted to a school leader/principal	1- Not at all likely<br>2- Not very likely<br>3 – Likely<br>4 - Very likely
		To pursue further education towards a degree outside of the field of education	
		To pursue further education towards a degree in education	
		To take a job outside of education	
		To attend to family responsibilities	
		To take a break from work	
		I will reach the retirement age as a teacher	
**Teacher-student Relations** and support to students	To what extent do you agree or disagree with the following statements?	If my students walked into my classes upset, I would be concerned about them.	1- Strongly disagree<br>2- Disagree<br>3- Agree<br>4- Strongly agree
		The students at my school are respectful towards me.	
		I provide individual help when a student has difficulties understanding a topic or task.	
		I tailor my teaching to meet the needs of my students	
		I am genuinely interested in how my students are doing	

Appendix 2
Semi-structured interview guide

- Can you describe your relationship with your students?
- How far do you enjoy the diverse and unexpected challenges that your role brings?
- To what extent do you believe that you have the necessary/formal training required to facilitate quality learning and address the diverse educational needs of the learners in your classroom?
- Describe your experience of job satisfaction
- To what level do you think you are committed to your work on a scale of 10? Why?
  0 = Not at all committed
  10 = Very committed
- How motivated are you to do your job on a scale of 10? Why?
- In your opinion, how do you think that teaching in the classroom impacts on your occupational well-being? (Cognitive, subjective, physical/mental and social wellbeing)
- With regards to organisational support, tell me how well the school supports you?
- Describe how you manage / cope with stress at work?
- How would you describe your relationships with leadership/management?
- How would you describe your dealings /relationships with colleagues?
- To what extent do you feel supported by your other colleagues and school administrators (other school stakeholders)?
- How many more years do you want to continue to work as a teacher?
- This school year, have you seriously considered leaving classroom teaching
